# Neuroprogression of chronic stress-induced depression via longitudinal fMRI

**DOI:** 10.3389/fpsyt.2026.1898694

**Published:** 2026-10-14

**Authors:** Yu Yang, Huixia Jia, He Liu

**Affiliations:** 1 Department of Systems Science, Faculty of Arts and Sciences, Beijing Normal University, Zhuhai, China; 2 International Academic Center of Complex Systems, Beijing Normal University, Zhuhai, China; 3 School of Systems Science, Beijing Normal University, Beijing, China

**Keywords:** chronic stress, depression, fMRI, neural network, neuronscience

## Abstract

**Background:**

Depression is increasingly viewed as a progressive neurobiological trajectory, yet the spatiotemporal transition from early mood dysregulation to chronic network failure, and its relation to disease-related functional decline, remains unclear. Using a chronic unpredictable mild stress (CUMS) mouse model, we aimed to map the longitudinal brain transformation from local spontaneous activity to global network dysfunction via multi-parametric MRI.

**Methods:**

Longitudinal functional magnetic resonance imaging (fMRI) was performed on a cohort of CUMS mice (n = 7) across three longitudinal time points: Day 0 (baseline), Day 5 CUMS, and Day 10 CUMS. We integrated four analytical approaches: fALFF, static functional connectivity, graph theory, and dFC/dALFF.

**Results:**

Depression progression followed a hierarchical spatiotemporal dissolution. fALFF revealed sequential breakdown of local hubs in caudoputamen, medial prefrontal cortex, and hippocampus, suggesting involvement of motor-motivational and executive deficits. sFC showed sensory-limbic uncoupling between caudoputamen and visual areas, and altered mPFC-AON connectivity and hippocampal prefrontal (HPF-PL) dysconnectivity, indicating limbic-olfactory loop disruption. Graph theory indicated preserved global node efficiency but widespread loss of node local efficiency and clustering. Dynamic analyses uncovered a shift from flexible network reconfiguration to dynamic rigidity.

**Conclusion:**

Longitudinal CUMS exposure was associated with progressive alterations in functional network organization. The prefrontal olfactory dysconnectivity and onset of dynamic rigidity represent candidate multi-scale and longitudinal imaging features that warrant future validation.

## Highlights

- Depression progression shows a staged pattern, moving from localized activity shifts to global network rigidity.
- Chronic stress primarily erodes local clustering and nodal efficiency while temporarily sparing the global connectome backbone.
- Progression toward severe depression is associated with reduced metastability, characterized by increased occupancy of a singular, low-connectivity functional state.
- The transition from diverse dynamic repertoires to temporal rigidity provides a mechanistic framework for understanding the temporal progression of chronic mood disorders.

## 1 Introduction

Major Depressive Disorder (MDD) is no longer viewed as a transient, reversible chemical imbalance but as a progressive neurobiological trajectory, a staged process characterized by cumulative structural and functional insults to the cerebral architecture (1, 2). In the context of chronic stress-induced pathology, this progressive deterioration is particularly relevant, as it intersects with systemic inflammation, oxidative stress and vascular vulnerability (3, 4). By the time severe symptoms manifest, the brain’s functional connectome has already undergone a potentially irreversible reorganization (5).

This suggests a silent period of neuroprogression where the brain remains behaviorally compensated despite underlying network decay (2, 6). For instance, theoretical models such as the disconnection syndrome suggest that progressive microstructural and neurovascular alterations can disrupt white matter tracts and metabolic pathways essential for emotional regulation (7, 8). Identifying the precise transition points, the tipping points, from a resilient state to a locked depressive state remains one of the most elusive goals in translational neuropsychiatry (9).

To understand the temporal progression of a depressive state, we must look at the hierarchy of brain failure, beginning at the voxel level. Local spontaneous fluctuations, measured via fractional amplitude of low frequency fluctuations (fALFF), reflect the regional intensity of low-frequency blood oxygen level-dependent (BOLD) oscillations in specific hubs like the medial prefrontal cortex (mPFC) and hippocampus (10, 11). In the early stages of depression, these regions often exhibit a paradoxical hyper-reactivity, a frantic attempt by neural tissue to maintain homeostatic output despite a declining synaptic density (12). However, this initial increase in spontaneous local activity eventually gives way to a secondary decline, potentially reflecting underlying neurovascular or cellular exhaustion under chronic stress (13, 14).

As these local signals falter, the disruption radiates outward, severing the functional connectivity (FC) across long range neural circuits. This decoupling is most pronounced in the reward circuit (striatal prefrontal) and the default mode network (DMN), where the synchronicity between the nucleus accumbens and higher order cortical regions begins to erode (15, 16). In the context of chronic stress, this decoupling may be further exacerbated by cumulative insults to myelin integrity, which increases conduction noise and reduces the signal-to-noise ratio of functional communication (17, 18). This shift from local changes to circuit level desynchronization may represent a critical transition from subclinical stress to overt moderate depression (19).

Beyond individual circuits, the global organization of the brain determines its overall resilience to pathological changes induced by stress. Using graph theory, we can treat the brain as a complex mathematical construct of nodes and edges (20). A healthy brain maintains a delicate balance between local specialized processing (clustering) and global integration (short path lengths), allowing for rapid information transfer while minimizing the metabolic cost of long distance wiring (21, 22).

However, chronic stress progressively impairs these topological properties. As depression reaches a severe stage, the “Rich Club” hubs, the high-traffic areas like the precuneus and anterior cingulate, begin to lose their central influence (23, 24). This leads to a breakdown of the small-world topology, characterized by an increase in characteristic path length (25). In this state, neural information must take a more circuitous and energy expensive route to travel across the brain, resulting in the cognitive slowing, executive dysfunction, and the psychomotor retardation that defines severe depressive states (26). This topological decay may reflect a loss of structural reserve and warrants further investigation as a candidate marker (27).

Perhaps the most critical, yet overlooked, aspect of the depressed brain is its dynamism. The healthy brain is not a static wiring diagram; it is a metastable system that fluidly transitions between various functional states to meet changing environmental demands (28, 29). These states represent transient patterns of co-activation that form and dissolve within seconds, a process governed by the brain’s entropic capacity (30).

In the CUMS model, this fluidity gives way to dynamic rigidity. By employing dynamic FC (dFC) and dynamic fALFF (dALFF), we can capture the flickering of these brain states (31, 32). This approach allows us to quantify the occupancy and dwell time of specific states. We hypothesize that as depression progresses, the brain becomes trapped in maladaptive, low-connectivity patterns, a sustained state of low functional connectivity and reduced global network integration (33, 34). This temporal rigidity mirrors the behavioral despair and anhedonia observed in mice and may explain why chronic depression becomes increasingly resistant to pharmacological treatment: the brain has lost the flexibility required to reconfigure its networks into a healthy state (35, 36).

To bridge these gaps, we utilized a longitudinal CUMS mouse model to simulate the longitudinal impact of chronic stress across three distinct time points: Day 0 (baseline), Day 5 CUMS, and Day 10 CUMS. By integrating local activity (fALFF), static network topology (Graph Theory), and temporal dynamics (dFC/dALFF), this study provides a comprehensive characterization of disease progression across the course of the disorder. We hypothesize that depression follows a hierarchical breakdown: beginning with local metabolic shifts, progressing to circuit-level decoupling, and culminating in a loss of global topological efficiency and temporal flexibility (2, 19, 37). By tracking these changes in a controlled longitudinal design involving seven mice (n = 7) scanned repeatedly across three time points, we provide a robust set of candidate imaging features that align with the neuroprogression framework, offering a new window of opportunity for stage specific therapeutic intervention prior to the onset of a persistent reduction in temporal network flexibility (38, 39). The graphical abstract of the present study is demonstrated in **Figure 1**.

**Figure 1:**
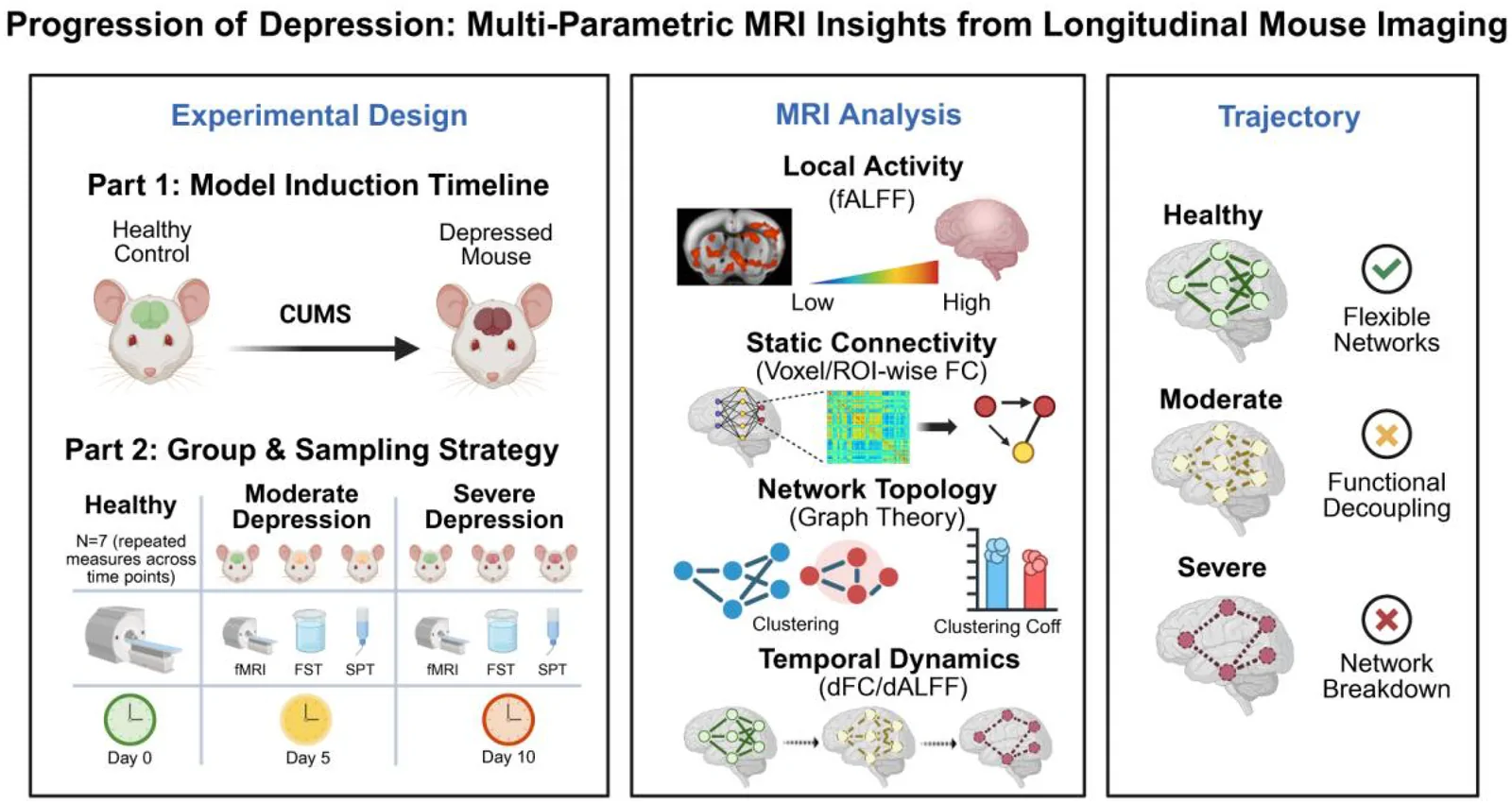
Graphical abstract of the present study.

## 2 Materials and methods

### 2.1 Animals depression model protocol

Adult male C57BL/6J mice were housed under a 12 h light/dark cycle with ad libitum access to food and water. All procedures were approved by the Beijing Normal University and performed in accordance with the NIH guide for the care and use of laboratory animals. To simulate the progressive nature of depression, mice underwent our improved CUMS protocol (**Table 1**) (35, 40). We increased the stress intensity by applying electric shock, restraint, tail clipping, and day-night reversal on a random schedule to ensure unpredictability (41). Specifically, each stressor was administered with the following parameters: restraint for 2 h; water deprivation for 6 h; food deprivation for 6 h; forced swimming for 10 min; tail clipping (pinching) for 10 min; cold exposure at 4 °C for 1 h; heat exposure at 45 °C for 10 min; day-night reversal for 12 h; cage tilting at 45°; and electric foot shocks (0.5 mA, 12 times within 10 min, delivered randomly). The order and combination of these stressors were pseudo-randomized across the 10-day protocol to avoid habituation. All mice were housed singly throughout the experiment to prevent social buffering effects, and the experimenters were blinded to group assignments during all behavioral and imaging procedures. Imaging was conducted at three distinct longitudinal milestones (2).

**Table 1:** The improved chronic unpredictable mild stress (CUMS) protocol.

Day	Morning	Afternoon	Night
0	**Imaging**		
1	Depriving Water Tilt	Clip Tail Restraint	Depriving Water & Food
2	Restraint Depriving Cushioning Materials	Low Temperature	Electric Shock Day-Night Reversal
3	High Temperature Depriving Food	Force Swimming Clip Tail	Tilt Restraint
4	Clip Tail Tilt	Restraint	Day-Night Reversal Electric Shock
5	**Imaging**		
6	Depriving Water Tilt	Clip Tail Electric Shock	Restraint
7	Restraint Depriving Food	High Temperature Force Swimming	Depriving Cushioning Materials Tilt
8	Depriving Water	Low Temperature Electric Shock	Restraint Tilt
9	Clip Tail Depriving Water & Food	Restraint Force Swimming	Electric Shock Day-Night Reversal
10	**Imaging**		

“Imaging” in bold refers to fMRI imaging.

All animals were subjected to the CUMS protocol, with no separate non-stressed control group. Instead, Day 0 (baseline) served as the within-subject control for each animal. A total of 10 mice were initially enrolled at the beginning of the study. Across the three imaging sessions, three mice died due to their inability to tolerate the stress associated with the imaging procedure (one mouse died following each of the Day 0, Day 5, and Day 10 scanning sessions). To ensure a strictly balanced longitudinal design, data from these three deceased animals were completely removed from all longitudinal analyses. Thus, the final cohort of animals that completed all three time points was n = 7, and this same cohort (n = 7) contributed to all analyses at Day 0, Day 5, and Day 10.

The three experimental time points were defined relative to CUMS exposure: Day 0 served as the baseline, Day 5 CUMS represented the mid-point of the 10-day stress protocol, and Day 10 CUMS represented the completion of the 10-day stress protocol. This staging approach follows the established CUMS literature, where progressive behavioral and neurobiological deterioration is observed over the course of chronic stress exposure (35, 41).

### 2.2 Data acquisition

Anaesthesia was induced with 2% isoflurane delivered in a mixture of 0.4 L/min medical air and 0.1 L/min O_2_. The animal was then positioned on an animal bed, and anaesthesia was maintained at 2% isoflurane. To reduce motion during scanning, the head was immobilized using ear bars, a bite bar, and a nose cone. Once the probe was secured within the scanner, the isoflurane concentration was decreased to 1.5%, administered in 0.4 L/min medical air and 0.1 L/min O_2_. Core body temperature was continuously monitored with a rectal thermometer (42). All physiological parameters remained within normal ranges during scanning (respiratory rate: 60–80 breaths/min; heart rate: 400–500 bpm). Structural and functional MRI images were acquired using Bruker BioSpec small animal MRI scanner system (9.4 T horizontal bore, 94/30 USR, Germany). Images were acquired using a T2-weighted TurboRARE sequence with the following parameters: repetition time (TR) = 2,000 milliseconds (ms), echo time (TE) = 15 ms, field of view (FoV) = 19.2 × 14.4 mm, matrix = 200 × 150, slice thickness = 0.4 mm, spacing between slices = 0.4 mm, flip angle = 90° and scanning time = 16 min (43). Rapid acquisition with refocused echoes (RARE) sequence was used to acquire T1-weighted images with the following parameters: RT = 3,000 ms, ET = 28 ms, FoV = 19.2 × 14.4 mm, matrix = 200 × 150, slice thickness = 0.4 mm, spacing between slices = 0.4 mm, flip angle = 90° and scanning time = 3 min (44).

### 2.3 Data preprocessing

Data preprocessing was performed using SPM12 (Wellcome Trust Centre for Neuroimaging, London, UK) and the GRETNA toolkit (45), supplemented by ANTs (Advanced Normalization Tools) (46) and ITK-SNAP for anatomical refinements (47). Given the small size of the rodent brain, all anatomical and functional images were first scaled by a factor of 10 to facilitate the use of algorithms optimized for human imaging. To synchronize the temporal acquisition of different slices, slice-timing correction was applied using sinc interpolation, resampling each slice’s BOLD time series to the mid-acquisition time (48). Functional images were then realigned using a 6-parameter rigid-body transformation to correct for head motion (49). To ensure high data quality, stringent exclusion criteria were applied: any subject with cumulative head motion exceeding 3 mm in displacement or 3° in rotation was excluded from further analysis (50). No additional subjects in the final longitudinal cohort (n = 7) were excluded during data preprocessing, as all remaining animals met the head motion thresholds. For each subject, the mean functional image was generated to serve as a reference for subsequent co-registration. To improve registration accuracy, manual brain extraction (skull stripping) was performed on both the mean functional and structural T_2_-weighted images using ITK-SNAP (47). Additionally, the origin of each scan was manually reoriented to ensure spatial consistency with the standard mouse brain template origin. Spatial normalization was achieved through a multi-step procedure using ANTs (46). First, the individual functional images were linearly co-registered to their corresponding structural images. Subsequently, the structural images were normalized to the standard mouse brain template space using both linear and non-linear (diffeomorphic) transformations. The resulting transformation matrices were then applied to the functional data, which were resampled to standard space. Finally, to improve the signal-to-noise ratio and reduce residual spatial noise, the normalized functional images were smoothed using a gaussian kernel of 4 × 4 × 4 mm^3^ (scaled space) full width at half maximum (FWHM) (51). To further minimize the influence of non-neural artifacts, several denoising steps were implemented. Linear detrending was applied to remove signal drift caused by scanner heating or physiological fluctuations. Nuisance covariates were regressed out using a multiple linear regression model, which included: (1) the Friston-24 motion parameters to account for complex head motion effects (49); (2) white matter (WM) signals; and (3) cerebrospinal fluid (CSF) signals (52). Finally, the data underwent band-pass temporal filtering (0.01-0.08 Hz) to isolate the frequencies of physiological interest and suppress high-frequency respiratory/cardiac noise and low-frequency fluctuations (53).

### 2.4 Neuroimaging metrics and connectivity analysis

To characterize the local spontaneous neural activity, the fALFF was calculated (10, 11). Consistent with standard fALFF protocols, the preprocessing for this metric excluded band-pass filtering to preserve the full frequency spectrum (11). The fALFF maps were calculated by dividing the power within the low-frequency range (0.01-0.08 Hz) by the total power across the entire frequency range (10). For group-level comparisons, these maps were standardized using m-transformation (dividing by the global mean) followed by z-score transformation. For voxel-wise FC, seed regions were defined based on the Allen Mouse Brain Common Coordinate Framework (CCFv3), including the cerebellum (54). Pearson’s correlation coefficients were computed between the mean time series of each ROI and the time series of every voxel in the rest of the brain. Subsequently, fisher’s r-to-z transformation was applied to the resulting FC maps to improve the normality of the distribution for statistical inference (55).

Functional brain networks were constructed using the allen brain atlas to define 391 anatomical regions of interest (ROIs) (54) (**Supplementary File**). To construct functional brain networks from the subject-level 391×391 Pearson correlation matrices, network topological properties were calculated using the graph theoretical network analysis toolkit (GRETNA v2.0.0). Specifically, to eliminate biologically ambiguous interpretations, negative correlation coefficients were set to zero, retaining positive-only edges. The resulting positive correlation matrices were transformed into binary unweighted graphs across a wide range of proportional network sparsity thresholds (S). Sparsity was defined as the ratio of existing edges to the total number of possible edges in the network. To ensure that network topology comparisons were independent of total edge count, a sparsity range of 0.05 ≤ S ≤ 0.40 with an incremental step of 0.01 was applied. For each sparsity threshold across this range, primary graph theoretical metrics were computed, including degree centrality (Dc), node efficiency (Ne), node clustering coefficient (NCc), and node local efficiency (NLe). For each metric, the area under the curve (AUC) across the sparsity continuum (S∈[0.05, 0.40]) was calculated to provide a single, threshold-independent scalar summarizing overall network topology. In cases of disconnected nodes resulting from sparse thresholding, nodes were retained in the graph structure, and path-length-based metric calculations (such as efficiency) were executed using standard GRETNA algorithms where the path length between disconnected pairs was assigned as infinity (1/∞ = 0), thus naturally accounting for network fragmentation without excluding isolated nodes (20, 55).

To characterize the temporal dynamics of brain activity and connectivity, a sliding window approach was implemented (31, 32).

1. Window characterization: For each subject, the preprocessed BOLD time series were segmented into 105 consecutive windows using a rectangular window profile with a window length of 30 TRs and a step size of 1 TR (56). This configuration was chosen to achieve a balance between capturing stable functional states and maintaining sufficient temporal resolution to detect rapid transitions in brain dynamics (31).
2. Metric calculation: dFC: Within each sliding window, a 391×391 functional connectivity matrix was constructed by calculating pairwise pearson’s correlation coefficients between the mean time courses of all ROIs. dALFF: Simultaneously, voxel-wise fALFF values were computed within each window to generate a sequence of dALFF maps, representing the spatial-temporal fluctuations of regional spontaneous activity over the 105 windows (32).
3. Temporal state clustering: All windowed dFC matrices and dALFF maps from all subjects and time points were concatenated. To identify recurrent brain states, a K-means clustering algorithm was applied using the city-block (Manhattan) distance metric (57). To determine the optimal number of clusters (*K*), we performed a heuristic search across a range of *K* = 2-5. To ensure the stability and reproducibility of the clustering results, the K-means algorithm was repeated 50 times with random initializations for each candidate K value. The optimal number of clusters was determined by the elbow criterion (within-cluster sum of squares) and confirmed by silhouette analysis. To resolve label-switching across iterations, cluster labels were aligned using the Hungarian algorithm before computing temporal properties. The resulting clusters were defined as quasi-stable brain states. Their temporal properties, including fractional time (the proportion of time spent in each state) and transition frequency (the number of times an individual switched between states), were extracted for further statistical comparison across time points (58).

For the temporal properties of the identified dFC and dALFF states (fractional time and transition frequency), group differences across the three time points were assessed using repeated measures ANOVA. The significance threshold for each state-level comparison was adjusted to α/K, where K is the number of states (K = 5 in the present study).

### 2.5 Statistical analysis

All voxel-based statistical analyses were performed using SPM12 (Statistical Parametric Mapping, Wellcome Trust Centre for Neuroimaging, London, UK) and the GRETNA toolkit (45), running on MATLAB (MathWorks, Natick, MA, USA). Data format conversion from DICOM to NIfTI was initially conducted via dcm2niix (59). To identify longitudinal changes in neural activity, a one-way repeated measures analysis of variance (ANOVA) was applied to the fALFF maps across the three time points. To ensure statistical rigor and minimize distribution assumptions, a non-parametric permutation test (5,000 permutations) was implemented within the GRETNA environment (60). The resulting F-statistical maps were thresholded at a voxel-level of p < 0.001 and a cluster-level of p < 0.05 to correct for multiple comparisons (61). Significant clusters were localized and visualized using xjview and MRIcroGL (62).

For clusters exhibiting a significant main effect of time, the mean fALFF values were extracted from the identified ROIs. For node-level graph theoretical metrics, repeated measures ANOVA was applied to identify regions showing significant longitudinal changes. To strictly control for false positive rates across the 391 anatomical ROIs, multiple comparison correction was implemented using the false discovery rate (FDR) approach (Benjamini-Hochberg procedure) applied across ROI-level tests for each graph metric independently (q < 0.05). Results surviving FDR correction are reported as statistically significant, whereas regions reaching uncorrected threshold (P < 0.05) are explicitly treated as exploratory findings and interpreted with due caution. All uncorrected p-values are reported alongside effect sizes for transparency. These extracted values were then used for *post-hoc* pairwise comparisons via paired-sample t-tests. To account for the inflation of type I error in multiple comparisons, p-values for the *post-hoc* tests were adjusted using the Šidák correction. A Šidák-adjusted P < 0.05 was considered statistically significant.

For edge-wise comparisons of whole-brain functional connectivity, repeated measures ANOVA was applied to each of the 391×391 functional connections across the three time points. Given the large number of edges tested, we controlled for multiple comparisons using the false discovery rate (FDR) correction. Specifically, an FDR threshold of q < 0.05 was applied to the resulting P-values across all edges to maintain the expected proportion of false positives at or below 5%. Only edges surviving this FDR correction were reported as significantly altered.

## 3 Results

### 3.1 Spatiotemporal evolution of local spontaneous activity

To identify the early to late physiological markers of depression progression, we first characterized the longitudinal shifts in local intrinsic brain activity using fALFF. A voxel-wise one-way ANOVA revealed 16 distinct brain clusters (**Table 2**) that exhibited significant fALFF alterations across the three experimental stages (**Figure 2**). These clusters were not randomly distributed but rather converged upon three critical functional anatomical systems: Caudoputamen (CP), mPFC and hippocampal formation (HPF).

**Table 2:** Voxel level spatial localization of differential nuclei.

Cluster	Brain region	Coordinates			F value	Cluster size
		X	Y	Z		
1	CP-R: Caudoputamen	1.325	0.15	-0.25	6.1283	29
2	SSs-L: Supplemental somatosensory area	-4.075	-0.65	1.15	6.8438	21
3	MOB-L: Main olfactory bulb	-1.475	4.55	0.55	4.7061	15
4	LSr: Lateral septal nucleus, rostral (rostroventral) part	-0.275	0.95	0.75	6.8363	24
5	COPY-R: Copula pyramidis	1.125	-7.25	1.55	6.2707	23
6	CP-L: Caudoputamen	-1.675	0.55	1.55	6.0901	18
7	MOB-R: Main olfactory bulb	5.25	3.35	1.75	8.7831	43
8	DG-L: Dentate gyrus	-1.275	-1.65	2.75	8.2916	34
9	ORB: Orbital area	-1.075	3.15	1.75	8.4576	26
10	SIM-L: Simple lobule	-2.275	-6.05	2.75	6.0895	16
11	POST-R: Postsubiculum	1.725	-3.45	3.35	5.8107	36
12	CA3-R: Field CA3	1.125	-1.45	2.55	7.6058	23
13	DG-L: Dentate gyrus	-1.875	2.85	2.75	4.3121	19
14	CUL: Culmen	-0.275	-5.85	3.35	9.652	23
15	Ice-L: Inferior colliculus, external	-1.675	-5.45	3.35	6.7535	24
16	SCs-L: Superior colliculus, sensory related	0.125	-4.05	3.75	7.2244	56

**Figure 2:**
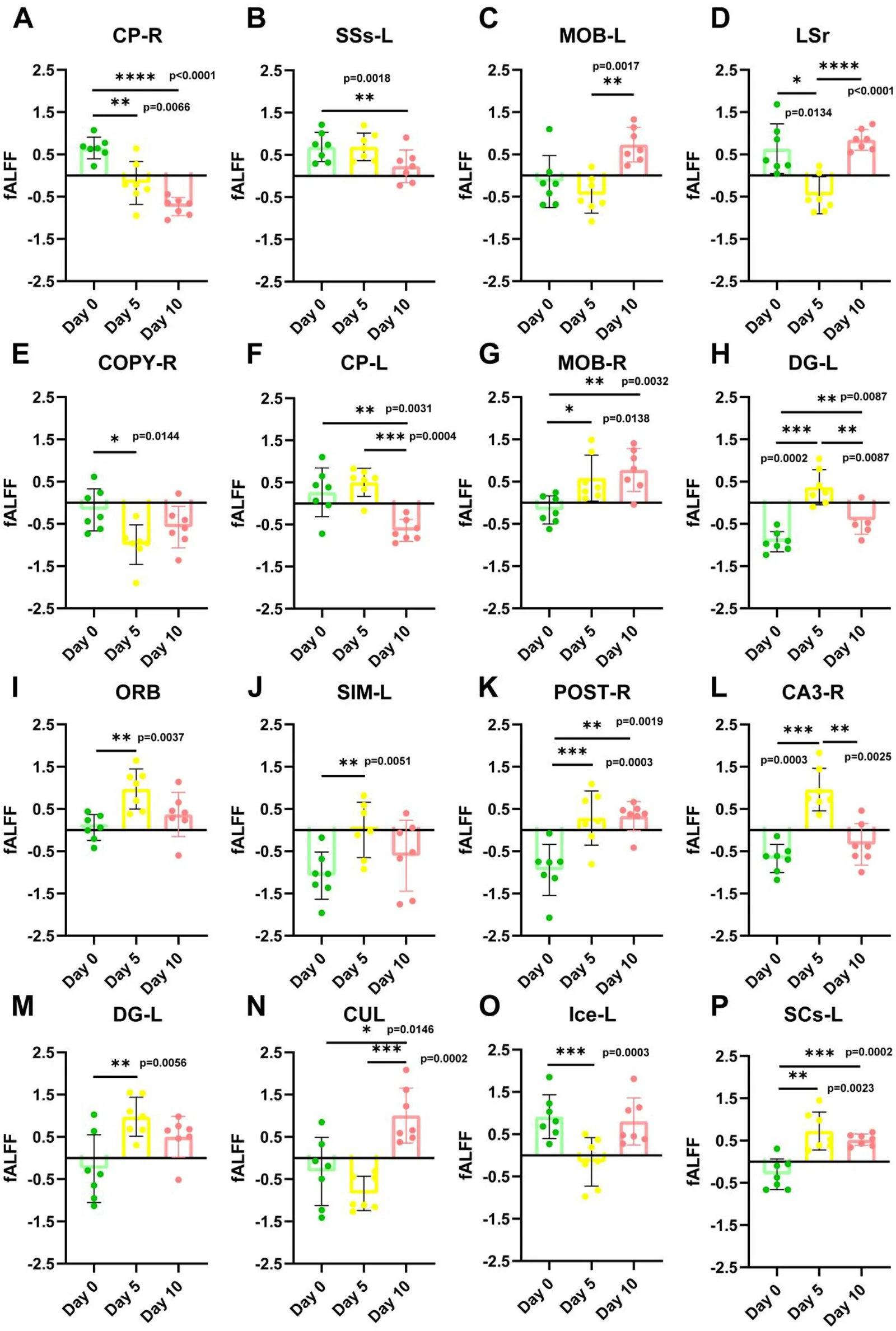
Differences in fALFF indicators among 16 brain regions (CP, caudoputamen; SSs, supplemental somatosensory cortex; MOB, main olfactory bulb; LSr, lateral septal nucleus, rostral part; COPY, copula pyramidis; DG, dentate gyrus; ORB, orbital cortex; SIM, simple lobule; POST, postsubiculum; CA3, hippocampal field CA3; CUL, culmen (cerebellar vermis); Ice, inferior colliculus, external nucleus; SCs, superior colliculus, sensory layer). These regions are subdivided into left (L) and right (R) sub-regions; Seven mice (n = 7) underwent repeated imaging at Day 0, Day 5, and Day 10; Data are expressed as mean ± SEM. Differences across time points (Day 0, Day 5, Day 10) were assessed by repeated measures ANOVA with Šidák correction for multiple comparisons at three different depression time (Day 0, Day 5 and Day 10) points **(A–P)**. Adjusted p-values: ****p ≤ 0.0001, ***p ≤ 0.001, **p ≤ 0.01, *p ≤ 0.05).

The striatal-motor interface (Caudoputamen): Significant fALFF variances were observed within the CP (F(2,12) = 6.13, *p* = 0.015, η_p_^2^ = 0.505; **Table 2**, cluster 1, 6). *Post-hoc* comparisons revealed a significant decrease in fALFF from Day 0 to Day 10 [t(6) = 4.23, P_Šidák_= 0.016, d = 1.38]. This progressive reduction in spontaneous activity within the CP, a region vital for sensorimotor and reward integration (63, 64), suggests a progressive disruption in the motor-motivation interface as CUMS exposure progressed from Day 5 to Day 10.

Top-down executive hubs (mPFC): The mPFC showed significant longitudinal changes in fALFF across the three time points (F(2,12) = 8.46, *p* = 0.005, η_p_^2^ = 0.585; **Table 2**, cluster 9). *Post-hoc* comparisons revealed a biphasic pattern: fALFF increased from Day 0 to Day 5 (t(6) = 2.98, P_Šidák_= 0.072, d = 0.96), followed by a significant decline from Day 5 to Day 10 [t(6) = -3.45, P_Šidák_= 0.04, d = 1.12]. This initial increase may reflect compensatory hyper-reactivity, whereas the subsequent decline may reflect a progressive impairment of top-down emotional regulation as CUMS exposure continued (65).

The stress vulnerable circuit (HPF): Analysis of the HPF revealed robust longitudinal differences (CA3-R: F(2,12) = 7.61, P = 0.008, η_p_^2^ = 0.559; DG-L: F(2,12) = 8.29; P= 0.006, η_p_^2^ = 0.58; **Table 2**, clusters 8, 12, 13). *Post-hoc* comparisons showed significant decreases in fALFF from Day 5 to Day 10 in both CA3-R [t(6) = -3.89, P_Šidák_= 0.024, d = 1.24] and DG-L [t(6) = -4.12, P_Šidák_=0.018, d=1.31]. Given the hippocampus’s sensitivity to glucocorticoid-mediated neurotoxicity and its role in cognitive-emotional integration (13, 66), these progressive decreases may reflect neural alterations associated with chronic stress exposure, which are commonly linked to cognitive and affective dysfunctions in rodent stress models.

Collectively, these localized fALFF changes indicate that the progression of depressive pathology is characterized by a hierarchical breakdown of spontaneous activity, beginning in localized functional hubs and eventually potentially compromising broader network synchrony.

### 3.2 Hierarchical breakdown of network connectivity

To investigate how localized fALFF alterations scale up to disrupt large scale brain communication, we performed seed-based voxel-wise FC analysis, using the previously identified hub regions (CP, mPFC, and HPF) as regions of interest.

#### 3.2.1 Striatal connectivity disruption

The CP seeded FC analysis revealed three distinct clusters exhibiting significant longitudinal remodeling across the depression trajectory (**Table 3**; **Figures 3A–C**).

**Table 3:** Seed-based functional connectivity mapping of the caudoputamen.

Brain region	Coordinates			F value	Cluster size
	X	Y	Z		
CP-R: Caudoputamen	1.525	0.35	0.35	20.8163	893
CP-L: Caudoputamen	-2.075	1.35	-0.05	16.1364	890
VISpm-R: posteromedial visual area	1.325	4.15	-3.05	27.6973	812

**Figure 3:**
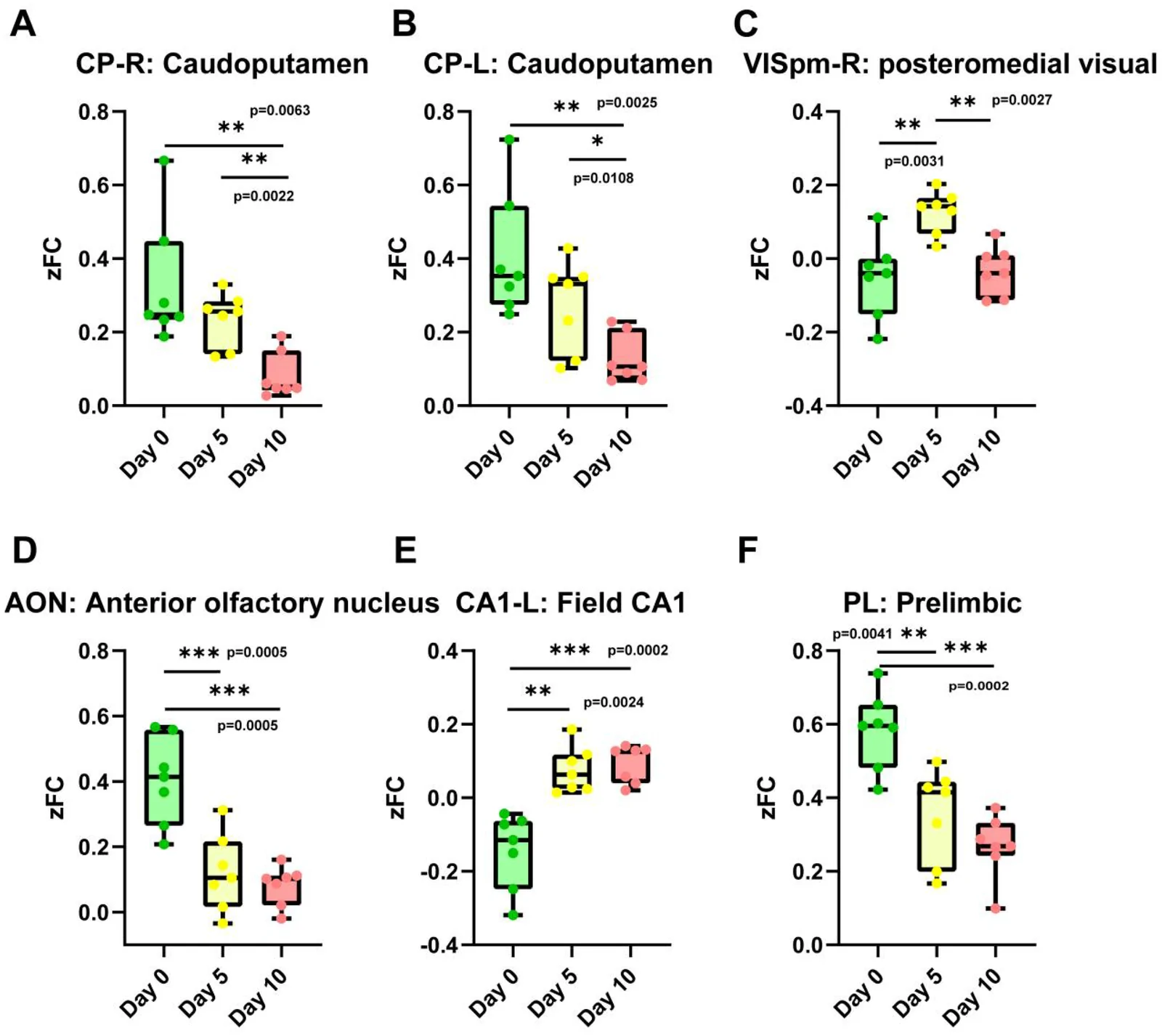
Longitudinal changes in seed-based voxel-wise functional connectivity across CUMS time points (Day 0, Day 5, and Day 10). **(A–C)** Caudoputamen (CP)-seeded functional connectivity; **(D)** Medial prefrontal cortex (mPFC)-seeded functional connectivity; **(E, F)** hippocampal formation (HPF)-seeded functional connectivity; Seven mice (n = 7) were scanned longitudinally across the three time points; Data are expressed as mean ± SEM. Differences across time points (Day 0, Day 5, Day 10) were assessed by repeated measures ANOVA with Šidák correction for multiple comparisons; Adjusted p-values: ***p ≤ 0.001, **p ≤ 0.01, *p ≤ 0.05.

Intra-striatal desynchronization: We observed significant FC changes in the bilateral caudoputamen (CP-R and CP-L). This loss of bilateral synchronization within the striatum may represent an early breakdown of the motor-motivation interface. Sensory-limbic uncoupling: Intriguingly, the CP showed progressive connectivity shifts with the Right Posteriomedial Visual Area (VISpm-R) (**Supplementary Figure S1A**). This uncoupling suggests that as depression worsens, the integration between primary sensory inputs and the reward-processing striatum becomes increasingly fragmented, a potential substrate for the “sensory blunting” often observed in chronic mood disorders (67, 68).

#### 3.2.2 Prefrontal olfactory and hippocampal prefrontal connectivity

The mPFC- and HPF-seeded analyses revealed connectivity changes involving the olfactory limbic interface and the hippocampal prefrontal pathway, respectively. Prefrontal AON circuitry: The mPFC seeded analysis identified a significant cluster in the AON (**Table 4**; **Figure 3D**; **Supplementary Figure S1B**). As the mPFC is a primary regulator of emotional top-down control (65), the altered connectivity with the AON highlights a disruption in the prefrontal-olfactory axis, which is closely linked to emotional memory and social recognition.

**Table 4:** Seed-based functional connectivity mapping of the prefrontal cortex.

Brain region	Coordinates			F value	Cluster size
	X	Y	Z		
AON: Anterior olfactory nucleus	-0.675	-0.05	2.55	24.6458	40

Hippocampal-prefrontal connectivity (HPF seed): The HPF-seeded analysis revealed two clusters showing significant longitudinal changes in functional connectivity, located in the left hippocampal field CA1 (CA1-L) and the prelimbic area (PL) (**Table 5****;** **Figures 3E, F**; **Supplementary Figure S1C**). The CA1 cluster indicates altered intra-hippocampal coupling, whereas the PL cluster points to altered connectivity along the hippocampal–prefrontal pathway. This pathway supports the integration of contextual and emotional information and is known to be vulnerable to chronic stress (66). Notably, the PL is a subdivision of the mPFC; the HPF-PL change therefore links the two hubs identified in the fALFF analysis. Together with the mPFC-AON finding, this suggests that CUMS exposure affects a broader prefrontal-hippocampal-olfactory network rather than isolated regions, consistent with reports of limbic and olfactory limbic dysfunction in clinical depression (69, 70).

**Table 5:** Hippocampus seed-based functional connectivity mapping.

Brain region	Coordinates			F value	Cluster size
	X	Y	Z		
CA1-L: Field CA1	4.125	1.95	-2.85	20.8163	62
PL: Prelimbic area	0.125	1.95	1.95	23.8485	115

### 3.3 Topometric reconfiguration and topological decay of the global connectome

To evaluate the systemic impact of depression progression on the brain’s organizational principles, we transitioned from voxel-wise circuits to an ROI based graph theoretical framework.

#### 3.3.1 Longitudinal shifts in ROI-wise functional edges

At the macroscopic level, the whole brain functional connectome exhibited significant remodeling. A total of 19 functional connections demonstrated distinct temporal trajectories as the depressive phenotype matured (**Figures 4A, B**; **Supplementary Figure S2**), suggesting that the progression of depression is not localized to a single circuit but represents a steady erosion of inter-regional synchrony across the entire connectome.

**Figure 4:**
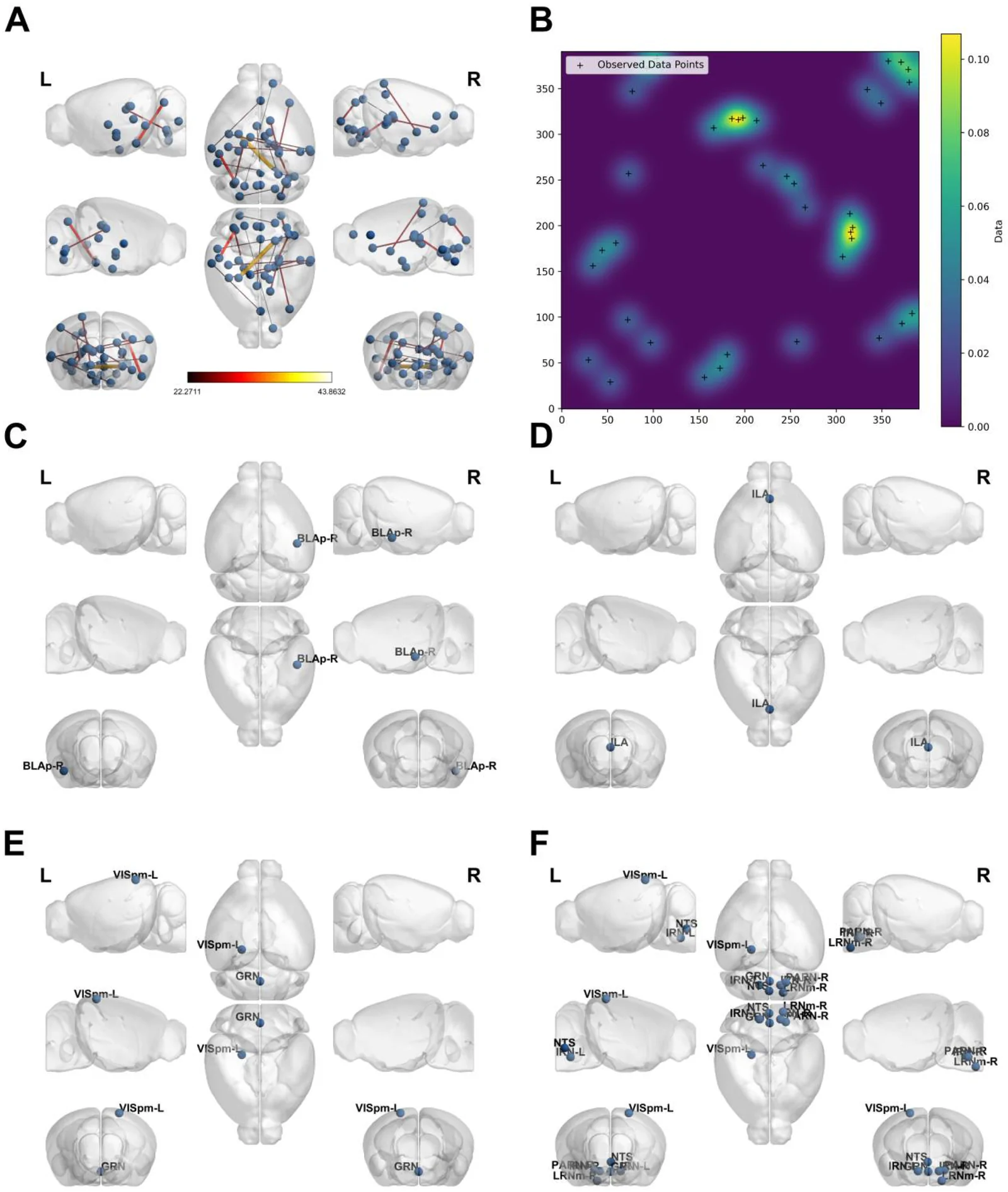
Whole-brain functional connectivity network and brain regions showing significant alterations in nodal topological properties. **(A)** Visualization of the whole-brain functional connectivity network. Blue spheres represent brain ROIs, and edges represent functional connections retained after graph thresholding. Edge colors indicate the strength of functional connectivity, with warmer colors representing stronger connections, as indicated by the color scale. **(B)** Heatmap showing the spatial distribution of functional connectivity strength/density. Black crosses indicate the observed data points, and the color scale represents the corresponding connectivity strength, with higher values shown in yellow. **(C–F)** Brain regions showing significant across time points differences in degree centrality **(C)**, node efficiency **(D)**, node clustering coefficient **(E)**, and node local efficiency **(F)**. Blue spheres indicate brain regions meeting the statistical significance criterion (P < 0.05), and anatomical labels identify the corresponding regions. The significant regions include posterior part of the basolateral amygdalar nucleus (BLAp-R) in **(C)**, infralimbic area (ILA) in **(D)**, visual cortex, posteromedial area (VISpm-L) and gigantocellular reticular nucleus (GRN) in **(E)**, and VISpm-L, nucleus of the solitary tract (NTS), intermediate reticular nucleus (IRN), parvicellular reticular nucleus (PARN-R), and lateral reticular nucleus (LRNm-R) in **(F)**. L and R indicate the left and right hemispheres, respectively.

#### 3.3.2 Hierarchical breakdown of nodal topology

The analysis of nodal topological metrics revealed a hierarchical pattern of impairment, where local segregation metrics were more sensitive to depression progression than global integration metrics. Only one region exhibited significant differences in Dc (**Figure 4C**; **Supplementary Figure S3A**) and Ne (**Figure 4D**; **Supplementary Figure S3B**). In contrast, the most pronounced alterations were observed in NLe and NCc. We identified 7 regions (VISpm-L, NTS, GRN, IRN-L/R, LRNm-R, PARN-R) with significant NLe changes [all F(2,12) > 6.50, *p* < 0.05] (**Figure 4F**; **Supplementary Figures S3E–K**) and two regions (GRN, VISpm-L) with significant NCc variances [F(2,12) = 7.21 and 6.89, respectively, *p* < 0.05] (**Figure 4E**; **Supplementary Figures S3C, D**). These findings indicate a progressive loss of cliquishness and local information processing capacity, suggesting that the brain’s modular resilience is severely compromised as the pathology moves from a moderate to a severe state. This topological pattern, characterized by localized reductions in NLe alongside a relatively preserved global network architecture, provides potential exploratory indicators for tracking stress-induced functional alterations.

### 3.4 Temporal dissolution and the emergence of dynamic rigidity

To capture the flickering nature of brain states and their erosion over time, we employed a sliding window approach combined with K-means clustering to quantify the temporal dynamics of local activity and global connectivity.

#### 3.4.1 Chronological transitions of local dynamic activity

K-means clustering of dALFF maps identified five distinct, recurring temporal states (States 1-5), capturing the brain’s of spontaneous regional activity (**Figure 5**; **Supplementary Figure S4**). Quantitative evaluation of dynamic state parameters revealed significant longitudinal alterations in temporal state metrics across the CUMS trajectory. Repeated measures ANOVA demonstrated a significant main effect of time on the fractional occupancy and dwell time of high-activity states (e.g., State 1: F(2,12) = 11.45, P_FDR_ = 0.003, η_p_^2^ = 0.656), which exhibited a progressive reduction over stress exposure. Conversely, fractional occupancy in low-activity states increased significantly over time (F(2,12) = 14.82, P_FDR_ = 0.002, η_p_^2^ = 0.712), accompanied by a significant overall decline in state transition frequency (F(2,12) = 8.76, η_p_^2^ = 0.594). These metrics indicate a restricted dynamic range of regional spontaneous fluctuations under chronic stress. Comprehensive quantitative details, including exact fractional occupancies, dwell times, transition matrices, *post-hoc* t-statistics, PFDR values, and effect sizes (Cohen’s d) across all states and time points, are provided in **Supplementary File**.

**Figure 5:**
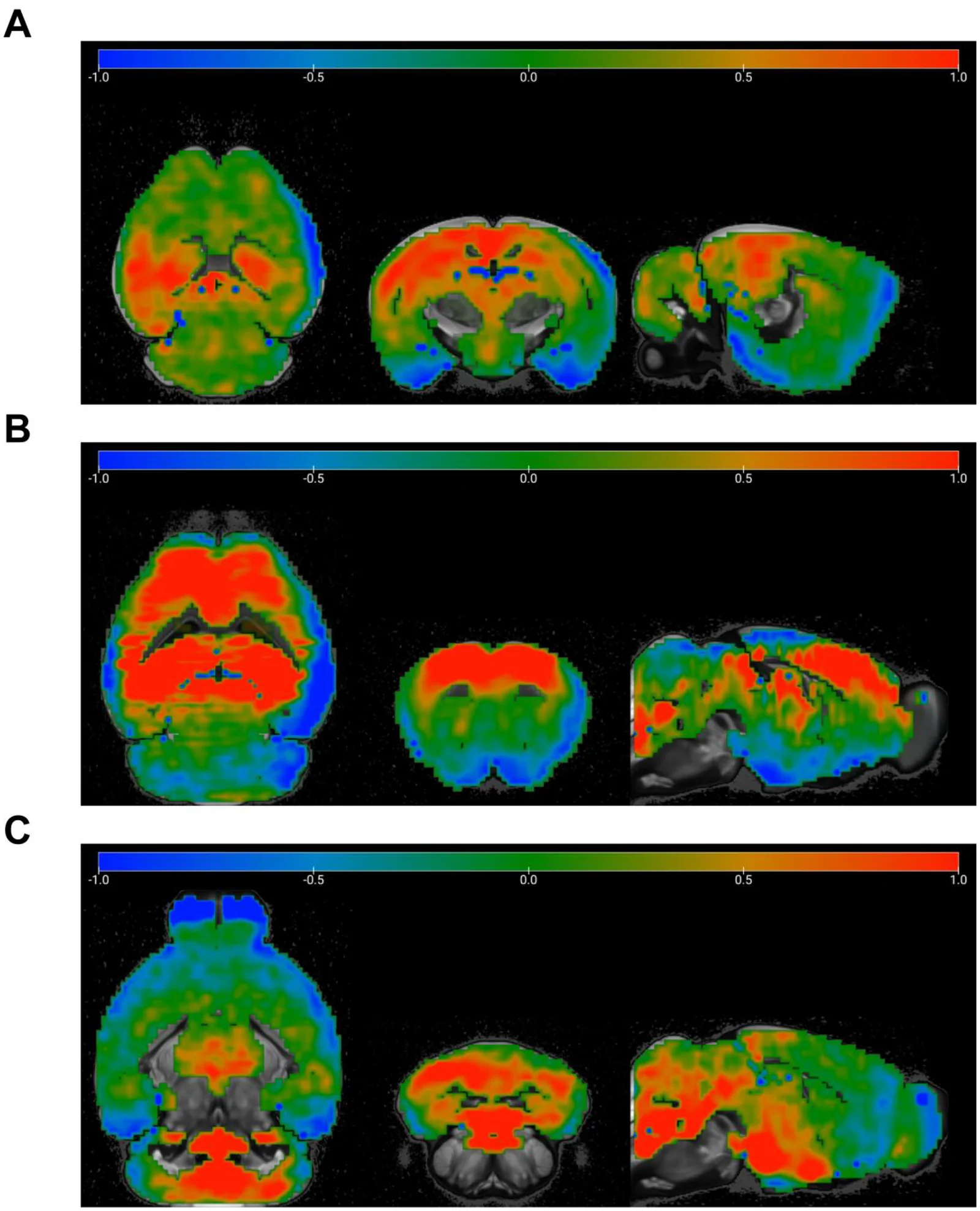
Representative brain states identified by dALFF clustering. State 1 **(A)**, State 3 **(B)**, and State 5 **(C)** are shown in the main figure as representative states selected in temporal order. The state maps were derived from the dALFF clustering analysis by averaging the dALFF maps assigned to each state. The color scale represents normalized dALFF values, ranging from -1 to 1, with warm colors indicating higher dALFF values and cool colors indicating lower dALFF values.

#### 3.4.2 Loss of network metastability

The dFC analysis further revealed alterations in the temporal organization of functional connectivity states during CUMS exposure. Visualization of the state assignments across successive time windows showed heterogeneous dFC patterns on Day 0 and Day 5, with multiple states represented across individual mice and time windows (**Figure 6A**). By Day 10, the temporal state-assignment pattern became more uniform, with State 3 visually occurring across many of the sampled time windows. This pattern indicates a reorganization of the dynamic connectivity landscape during prolonged stress exposure; however, state occupancy and transition frequency were not quantitatively evaluated in the present analysis. The corresponding state-specific functional connectivity matrices showed distinct connectivity patterns among the three identified states (**Figures 6B–D**). State 1 exhibited relatively widespread positive ROI-ROI connectivity, State 2 showed an intermediate connectivity pattern, whereas State 3 was characterized by generally weaker pairwise functional connectivity. Together, these observations indicate that prolonged CUMS exposure was associated with changes in both the temporal organization and connectivity profiles of dFC states, with the later stage displaying a greater representation of a low-connectivity configuration. This pattern is consistent with previous reports of altered network dynamics under prolonged stress (71).

**Figure 6:**
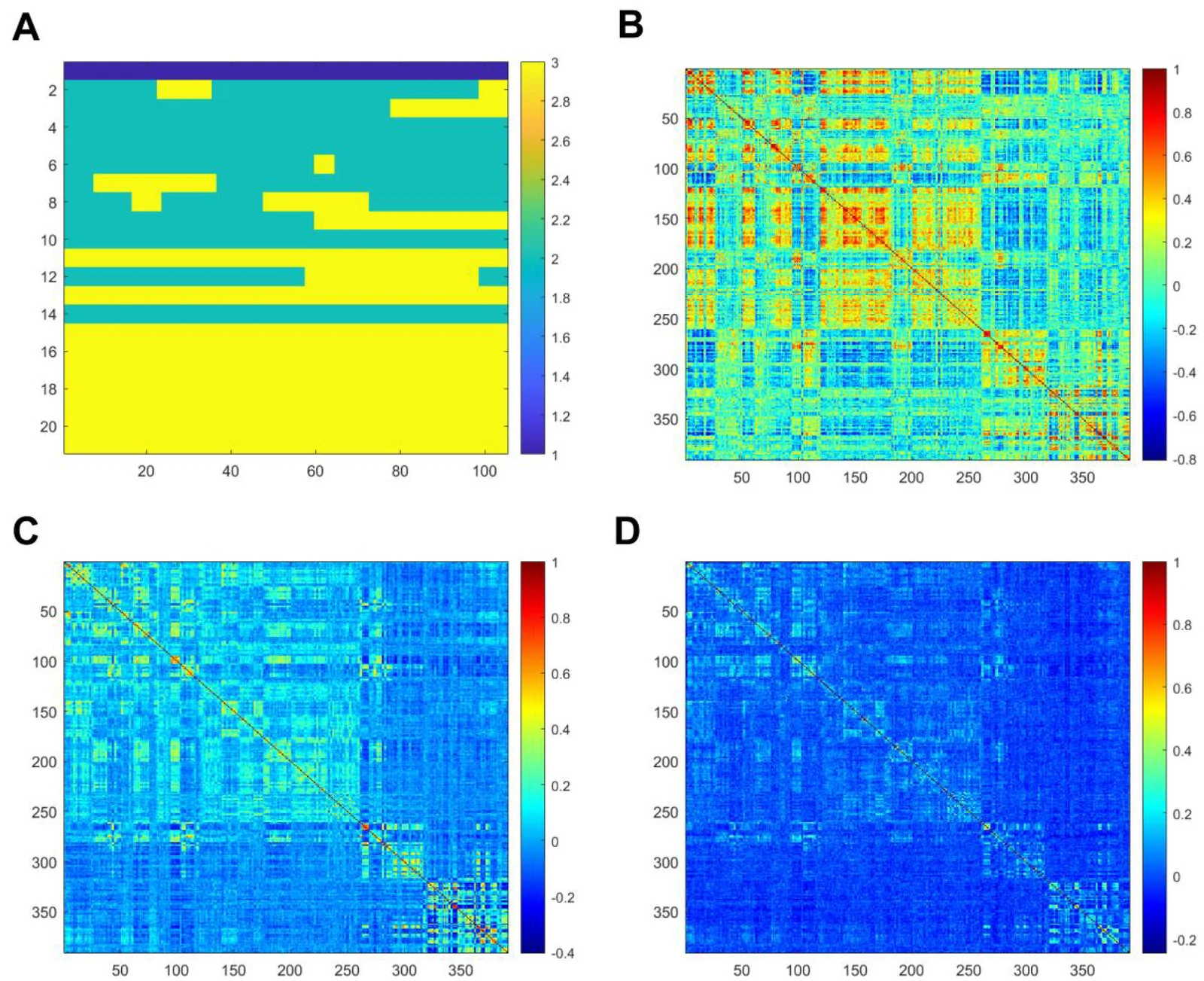
Temporal distribution and representative connectivity patterns of dFC states across mice. **(A)** State assignments obtained from dFC clustering across successive time windows for individual mice. Each row represents one mouse, with rows 1–7 corresponding to Day 0, rows 8–14 to Day 5, and rows 15–21 to Day 10. Each column represents one time window. Colors in panel A indicate the cluster/state assigned to each time window and represent categorical state labels rather than connectivity magnitude. **(B–D)** Representative functional connectivity matrices corresponding to the three dFC states identified by clustering. Each matrix shows the connectivity pattern between brain regions for the corresponding state. The color scale represents FC values, with warmer colors indicating higher positive connectivity values and cooler colors indicating lower or negative connectivity values.

## 4 Discussion

In this study, Day 0 baseline measurements served as a within subject control, allowing each animal to act as its own baseline across the longitudinal CUMS trajectory. While this design minimizes inter-individual variability and enhances statistical power for tracking dynamic trajectory shifts, it cannot fully disentangle CUMS-specific neurobiological stress effects from secondary longitudinal influences. These confounders potentially include repeated anesthesia, repeated MRI gradient/radiofrequency exposure, habituation to handling, single housing, repeated behavioral testing, and time-dependent aging processes. To minimize these influences, an identical anesthesia protocol (2% isoflurane for induction and 1.5% for maintenance) with continuous physiological monitoring and identical acquisition parameters was used at every session, no stressors were applied on imaging days (**Table 1**), and the entire protocol spanned only 10-day in adult animals, limiting potential age-related effects. Consequently, the observed functional network shifts should be interpreted as alterations associated with the overall longitudinal CUMS experimental trajectory rather than strictly isolated stress-specific effects. Future longitudinal studies incorporating a parallel, non-stressed control cohort scanned at identical time intervals will help isolate these specific components.

Our findings suggest that the 10 days of depression in mice is a quintessential example of allostatic overload, that is, cumulative wear and tear on the brain’s regulatory systems (72, 73). These findings are consistent with the concept of reduced metastability in complex systems. This low-connectivity state represents a transition from a dynamic, adaptive system to a highly constrained functional configuration, which theoretically parallels a low-entropy state in network dynamics (33, 34). In complex systems research, this loss of complexity is a hallmark of functional decline (74, 75). The transition from baseline state to a singular, dominant State 3 at the severe stage suggests a reduction in network flexibility, making it unable to generate the complex neural patterns required for resilient emotional regulation (29, 71).

The 16 clusters of altered fALFF include CP, mPFC, and HPF, suggesting region-specific alterations in spontaneous BOLD activity that may reflect underlying neurobiological changes (4, 76–79). The observed fALFF trajectory demonstrates a stage-dependent change in spontaneous low frequency BOLD activity. Its metabolic, cellular, and neurovascular basis cannot be determined from the present study.

One of our most novel findings is the involvement of the AON and VISpm in voxel level FC analysis. The AON serves as a primary limbic sensory gatekeeper (81). Olfactory-limbic pathways are known to be particularly sensitive to network breakdown across various chronic psychiatric and neurodegenerative conditions (69, 80). Our findings demonstrate that stress-induced depression directly engages these vulnerable sensory-limbic loops in adult mice, highlighting an important circuit node for future studies examining stress-related neuroprogression. The CP-VISpm decoupling suggests a failure in bottom-up sensory integration (82). As the brain becomes less responsive to external visual and olfactory cues, this may serve as a protective mechanism to reduce allostatic load. Ultimately, however, it isolates the brain, reinforcing depressive rumination and behavioral despair (68, 83).

Our graph theory results reveal a constrained network architecture prioritizing localized metabolic efficiency over global dynamic integration. The brain must balance the metabolic cost of connections with the functional need for integration (21, 22). The stability of node efficiency and betweenness suggests that the mouse brain prioritizes the structural skeleton of the connectome (23, 84). This may be an adaptive strategy whereby the brain cannot afford to lose its global communication pathways. However, the widespread decline in node local efficiency (NLe) across 7 brain regions indicates that functional modules are being compromised (25, 85). The brain appears to prioritize the preservation of global integration at the expense of local specialized processing (86, 87). Our findings suggest that the hallmark of the functionally deteriorating brain may be a loss of modular complexity, rather than a total breakdown of communication (27, 88). We speculate that this could relate to the concept of cognitive reserve. The transition to State 3 in our dFC analysis at day 10 may reflect a critical shift in network dynamics. Visualization of State 3 shows a global weakening of interactions, suggesting a marked reduction in network flexibility. In system dynamics, this is known as an extended relaxation time in network fluctuation patterns, which is conceptually analogous to critical slowing down phenomena observed prior to state transitions in complex systems (89, 90).

The network alterations observed in our longitudinal model provide a mechanistic framework for understanding chronic stress-induced neuroprogression. Because all metrics used here are derived from non-invasive, task-free resting-state fMRI, these features could, if validated, inform the development of imaging-based tools for the early detection and staging of depression in humans. Such validation will require independent rodent cohorts including sham-control animals, both sexes and behavioral phenotyping, as well as longitudinal human resting-state fMRI cohorts, such as first-episode, drug-naive patients and individuals at high risk for depression. While our study was conducted in adult mice, these multi-scale imaging features (e.g., AON dysconnectivity and dynamic network rigidity) may represent shared circuit vulnerabilities that could have broader translational relevance, including potential applications in studying mood disorders across different stages of adulthood. Future studies incorporating aged animal cohorts will be necessary to directly test how age-related baseline changes interact with stress-induced connectome deterioration.

Future research should integrate diffusion tensor imaging (DTI) and post-mortem histology (e.g., GFAP, Iba1 and MBP staining for astrocytes, microglia and myelin, respectively) to assess whether the dynamic rigidity we observed is purely functional or is underpinned by cellular and white matter alterations (17, 91).

## 5 Conclusion

Our study demonstrates that the progression of depression is a multi-scale unraveling of the brain’s functional architecture. It begins as a localized functional alteration, spreads as a circuit-level sensory-limbic disconnection, and terminates as a global topological and temporal rigidity. By identifying the prefrontal-olfactory dysconnectivity and state switching, we identify candidate imaging targets that move beyond simple connectivity measures and warrant future validation and toward a truly dynamic, staging-based approach to neuropsychiatric health.

## Data Availability

The original contributions presented in the study are included in the article/**Supplementary Material**. Further inquiries can be directed to the corresponding author.

## Supplementary material

Supplementary Figure 1 Visualized seed point location: CP **(A)**, mPFC **(B)** and hippocampal formation **(C).**

Supplementary Figure 2 The 19 functional connections in the whole brain functional connectivity (A–S). AUDp, posterior part of the primary auditory cortex; ILA, infralimbic area; PERI, perirhinal cortex; CA2, hippocampal field CA2; VISal, visual cortex, anterolateral area; CEAc, central amygdalar nucleus, lateral capsular part; VISpm, visual cortex, posteromedial area; BST, bed nucleus of the stria terminalis; AIv, agranular insular cortex, ventral part; PoT, posterior thalamic nuclear group; VMH, ventromedial hypothalamic nucleus; PeF, perifornical nucleus; ECT, ectorhinal cortex; TU, olfactory tubercle; PF, parafascicular nucleus; Ice, inferior colliculus, external nucleus; GPi, globus pallidus, internal segment; P5, layer 5 of the pons; PT, parategmental nucleus of the thalamus; RPO, rostral pontine reticular nucleus; LP, lateral posterior thalamic nucleus; DCO, dorsal cochlear nucleus; MGm, medial geniculate nucleus, medial part; Eth, ethmoid nucleus; VCO, ventral cochlear nucleus; AOB, accessory olfactory bulb; PARN, parvicellular reticular nucleus; GRN, gigantocellular reticular nucleus; PGRNd, paragigantocellular reticular nucleus, dorsal part; TR, trigeminal spinal tract nucleus, rostral part; SIM, simple lobule; COPY, copula pyramidis; SPIV, spinal trigeminal nucleus, interpolar part; ENT, entorhinal cortex; FL, flocculus; Each nucleus is subdivided into left (L) and right (R) subregions; Seven mice (n = 7) were scanned repeatedly across time points; Data are expressed as mean ± SEM. Differences across time points (Day 0, Day 5, Day 10) were assessed by repeated measures ANOVA with Šidák correction for multiple comparisons; Adjusted p-values: ****P ≤ 0.0001, ***p ≤ 0.001, **p ≤ 0.01, *p ≤ 0.05.

Supplementary Figure 3 The significant differences in graph theory node attributes among different brain regions: Degree centrality **(A)**, node efficiency **(B)**, node clustering coefficient **(C, D)** and node local efficiency **(E–K)**; Seven mice (n = 7) were scanned repeatedly across time points; Data are expressed as mean ± SEM. Differences across time points (Day 0, Day 5, Day 10) were assessed by repeated measures ANOVA with Šidák correction for multiple comparisons; Adjusted p-values: ****p ≤ 0.0001, ***p ≤ 0.001, **p ≤ 0.01, *p ≤ 0.05.

Supplementary Figure 4 dALFF clustering of different brain states, state 2 **(A)** and state 4 **(B)**; Red indicates higher ALFF values (more active brain regions), and blue indicates lower dALFF values (less active brain regions).
