# Supplementary material for: Neuroprogression of chronic stress-induced depression via longitudinal fMRI
Source: Front Psychiatry. 2026 Oct 14;17:1898694. doi: 10.3389/fpsyt.2026.1898694 (PMC13714589; doi:10.3389/fpsyt.2026.1898694)
Supplement: Supplementary file 1 [file DataSheet1.pdf]

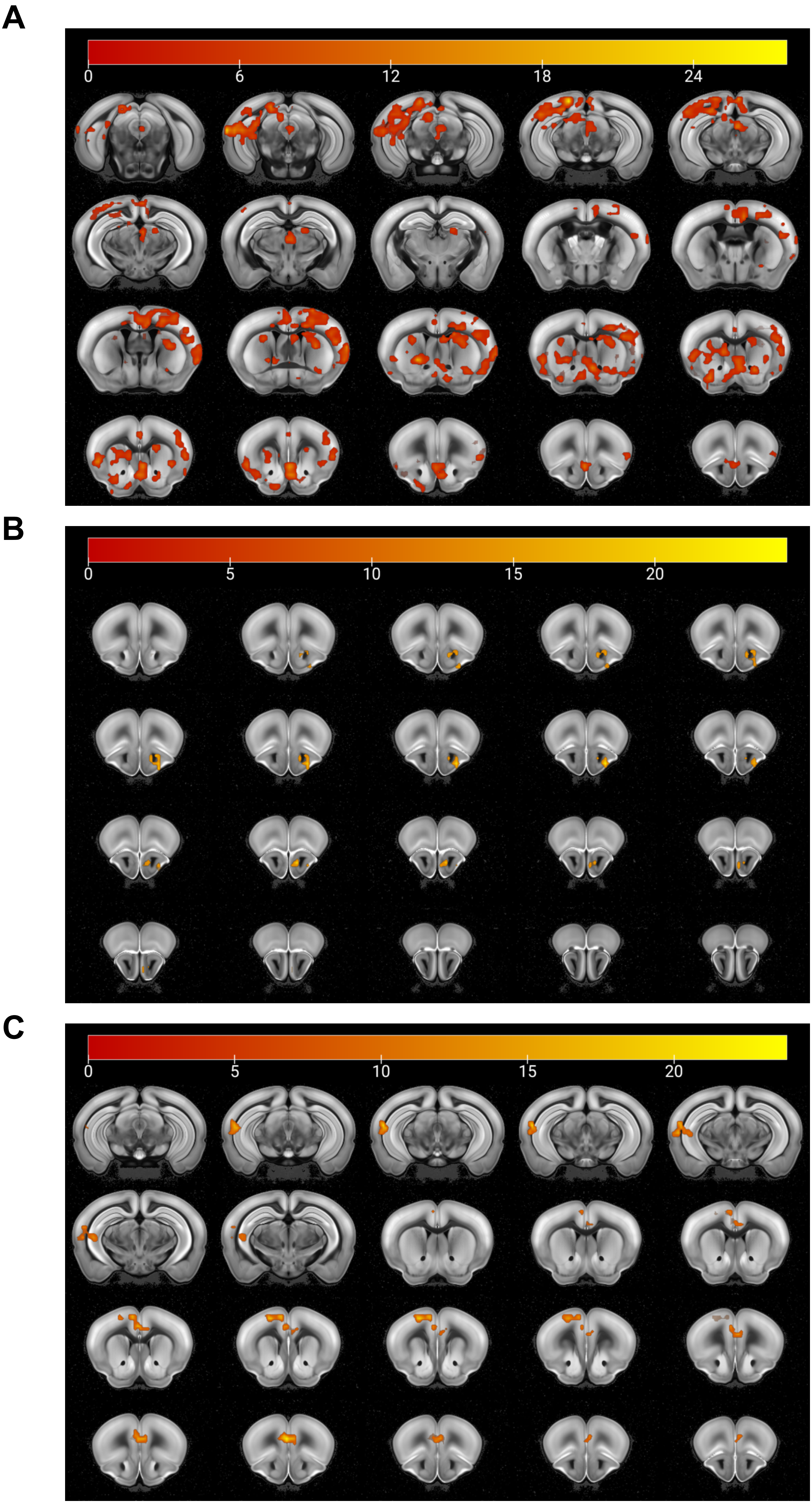
A
0
6
12
18
24
B
0
5
10
15
20
C
0
5
10
15
20
