# Supplementary material for: Neuroprogression of chronic stress-induced depression via longitudinal fMRI
Source: Front Psychiatry. 2026 Oct 14;17:1898694. doi: 10.3389/fpsyt.2026.1898694 (PMC13714589; doi:10.3389/fpsyt.2026.1898694)
Supplement: Supplementary file 2 [file DataSheet2.pdf]

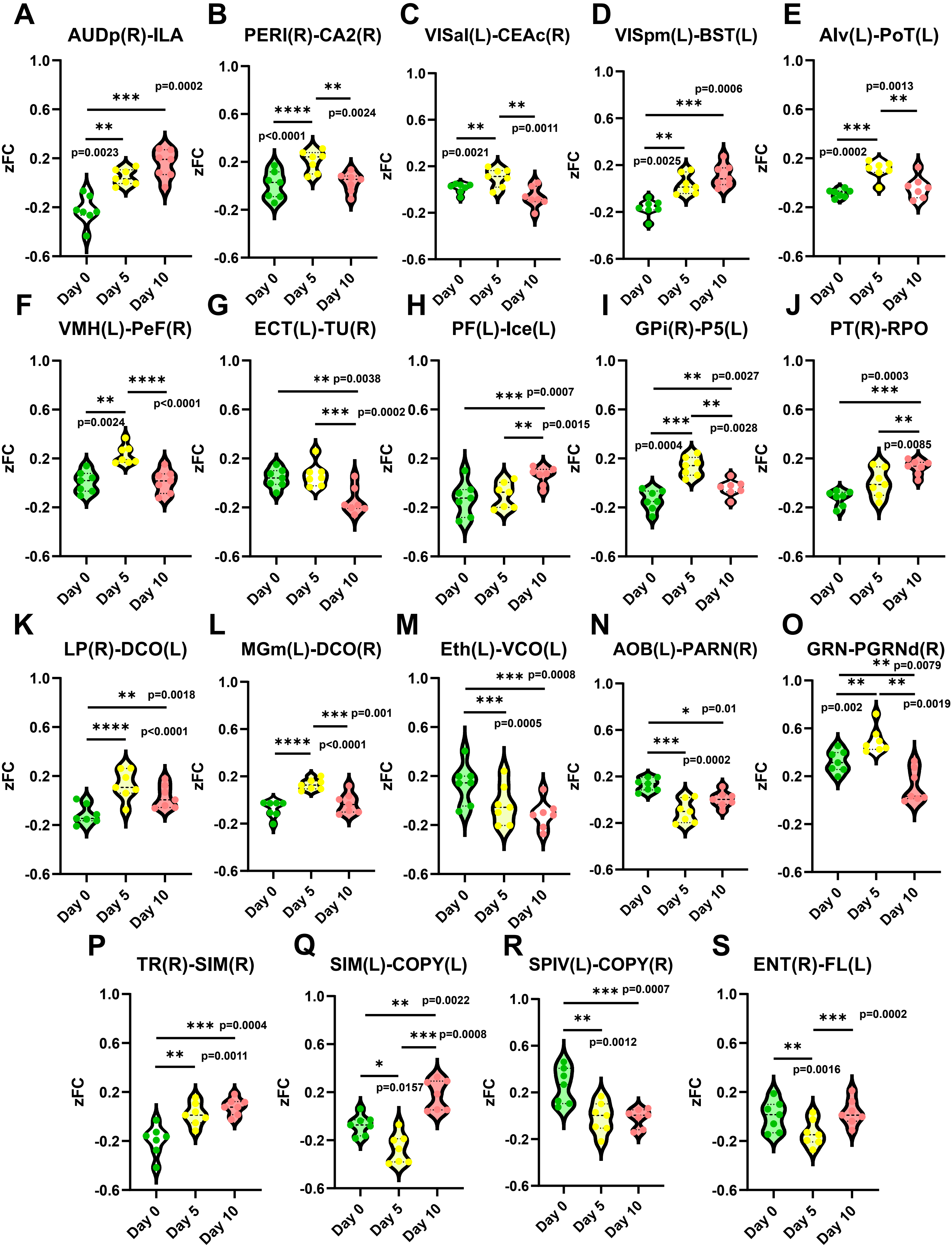

A
AUDp(R)-ILA
B
PERI(R)-CA2(R)
C
VISal(L)-CEAc(R)
D
VISpm(L)-BST(L)
E
AIv(L)-PoT(L)
F
VMH(L)-PeF(R)
G
ECT(L)-TU(R)
H
PF(L)-Ice(L)
I
GPi(R)-P5(L)
J
PT(R)-RPO
K
LP(R)-DCO(L)
L
MGm(L)-DCO(R)
M
Eth(L)-VCO(L)
N
AOB(L)-PARN(R)
O
GRN-PGRNd(R)
P
TR(R)-SIM(R)
Q
SIM(L)-COPY(L)
R
SPIV(L)-COPY(R)
S
ENT(R)-FL(L)
zFC
Day 0
Day 5
Day 10
p=0.0023
p=0.0002
p<0.0001
p=0.0024
p=0.0021
p=0.0011
p=0.0025
p=0.0006
p=0.0002
p=0.0013
p<0.0001
p=0.0038
p=0.0007
p=0.0015
p=0.0004
p=0.0027
p=0.0028
p=0.0003
p=0.0085
p=0.0018
p=0.001
p=0.0008
p=0.0005
p=0.01
p=0.002
p=0.0079
p=0.0019
p=0.0004
p=0.0011
p=0.0022
p=0.0157
p=0.0012
p=0.0016
