# Supplementary material for: Neuroprogression of chronic stress-induced depression via longitudinal fMRI
Source: Front Psychiatry. 2026 Oct 14;17:1898694. doi: 10.3389/fpsyt.2026.1898694 (PMC13714589; doi:10.3389/fpsyt.2026.1898694)
Supplement: Supplementary file 3 [file DataSheet3.pdf]

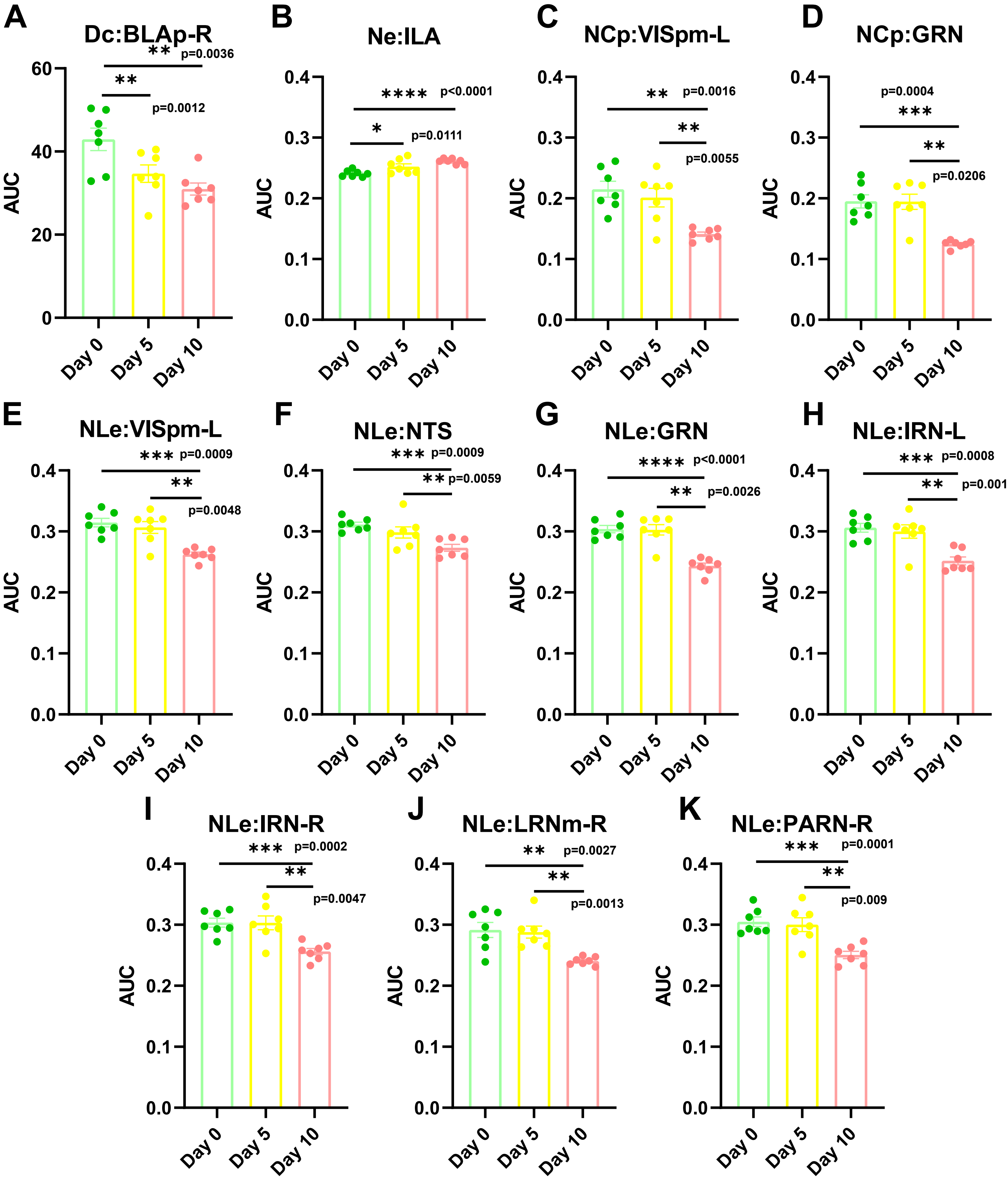

A
Dc:BLAp-R
** p=0.0036
**
p=0.0012
AUC
60
40
20
0
Day 0
Day 5
Day 10
B
Ne:ILA
**** p<0.0001
*
p=0.0111
AUC
0.4
0.3
0.2
0.1
0.0
Day 0
Day 5
Day 10
C
NCp:VISpm-L
** p=0.0016
**
p=0.0055
AUC
0.4
0.3
0.2
0.1
0.0
Day 0
Day 5
Day 10
D
NCp:GRN
p=0.0004
***
**
p=0.0206
AUC
0.4
0.3
0.2
0.1
0.0
Day 0
Day 5
Day 10
E
NLe:VISpm-L
*** p=0.0009
**
p=0.0048
AUC
0.4
0.3
0.2
0.1
0.0
Day 0
Day 5
Day 10
F
NLe:NTS
*** p=0.0009
** p=0.0059
AUC
0.4
0.3
0.2
0.1
0.0
Day 0
Day 5
Day 10
G
NLe:GRN
**** p<0.0001
** p=0.0026
AUC
0.4
0.3
0.2
0.1
0.0
Day 0
Day 5
Day 10
H
NLe:IRN-L
*** p=0.0008
** p=0.001
AUC
0.4
0.3
0.2
0.1
0.0
Day 0
Day 5
Day 10
I
NLe:IRN-R
*** p=0.0002
**
p=0.0047
AUC
0.4
0.3
0.2
0.1
0.0
Day 0
Day 5
Day 10
J
NLe:LRNm-R
** p=0.0027
**
p=0.0013
AUC
0.4
0.3
0.2
0.1
0.0
Day 0
Day 5
Day 10
K
NLe:PARN-R
*** p=0.0001
**
p=0.009
AUC
0.4
0.3
0.2
0.1
0.0
Day 0
Day 5
Day 10
