# Supplementary material for: Neuroprogression of chronic stress-induced depression via longitudinal fMRI
Source: Front Psychiatry. 2026 Oct 14;17:1898694. doi: 10.3389/fpsyt.2026.1898694 (PMC13714589; doi:10.3389/fpsyt.2026.1898694)

A

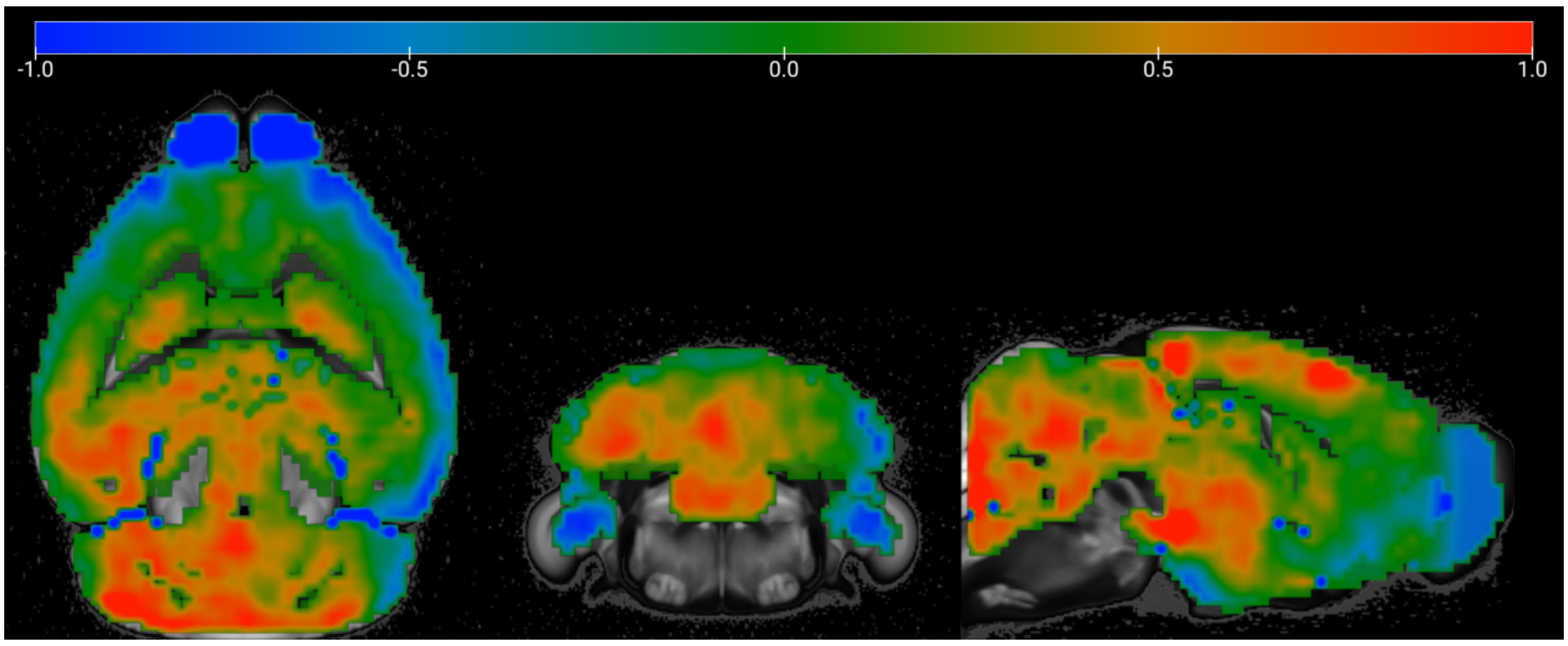

B

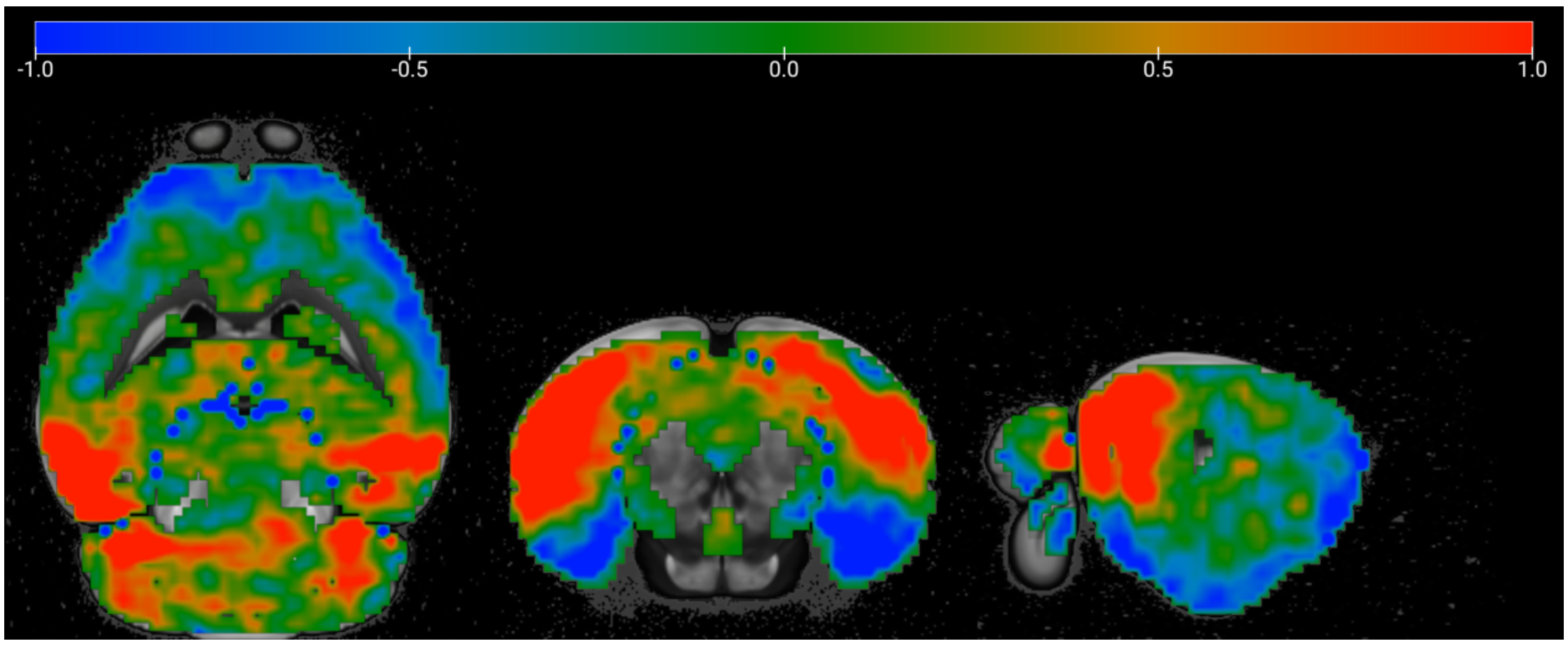

Supplement: Supplementary file 4 [file DataSheet4.pdf]
